# GT1b-induced neurotoxicity is mediated by the Akt/GSK-3/tau signaling pathway but not caspase-3 in mesencephalic dopaminergic neurons

**DOI:** 10.1186/1471-2202-11-74

**Published:** 2010-06-12

**Authors:** Eun S Chung, Eugene Bok, Sunghyang Sohn, Young D Lee, Hyung H Baik, Byung K Jin

**Affiliations:** 1 Department of Biochemistry and Molecular Biology, School of Medicine Kyung Hee University, Seoul 130-701, Korea; 2 Neurodegeneration Control Research Center, Age-related and Brain Diseases Research Center, School of Medicine Kyung Hee University, Seoul 130-701, Korea; 3 Brain Disease Research Center, Neuroscience Graduate Program, Division of Cell Transformation and Restoration, Department of Pharmacology, Ajou University School of Medicine, Suwon, 442-721, Korea; 4 Laboratory Cell Biology, Institute for Medical Sciences, Ajou University School of Medicine, Suwon, 442-721, Korea; 5 Department of Anatomy, Ajou University School of Medicine, Suwon, 442-721, Korea

## Abstract

**Background:**

Gangliosides, sialic acid-containing glycosphingolipids exist in mammalian cell membranes particularly neuronal membranes. The trisialoganglioside (GT1b) is one of the major brain gangliosides and acts as an endogenous regulator in the brain. We previously showed GT1b induces mesencephalic dopaminergic (DA) neuronal death, both *in vivo *and *in *vitro. We further investigate the underlying mechanisms of GT1b neurotoxicity.

**Results:**

Consistent with earlier findings, GT1b attenuated the DA neuron number and dopamine uptake level in mesencephalic cultures. Morphological evidence revealed GT1b-induced chromatin condensation and nuclear fragmentation as well as an increased number of TUNEL-positive cells, compared to control cultures. Interestingly, while GT1b enhanced caspase-3 activity, DEVD, a caspase-3 inhibitor, failed to rescue DA neuronal death. Immunoblot analysis revealed that GT1b inactivates Akt through dephosphorylation at both Ser473 and Thr308, subsequent dephosphorylation of GSK-3β, a substrate of Akt, and hyperphosphorylation of tau, downstream of GSK-3β. Moreover, a GSK-3β specific inhibitor, L803-mt, attenuated tau phosphorylation and rescued DA neurons from cell death in mesencephalic cultures.

**Conclusion:**

Our data provide novel evidence that a Akt/GSK-3β/tau-dependent, but not caspase-3 signaling pathway plays a pivotal role in GT1b-mediated neurotoxic actions on mesencephalic DA neurons.

## Background

The progressive degeneration of dopaminergic (DA) neurons in the substantia nigra (SN) is a well-known characteristic of Parkinson's disease (PD). *In vivo *and *in vitro *models of PD induced by 1-methyl-4-phenyl-1,2,3,6-tetrahydropyridine (MPTP), 1-methyl-4-phenylpyridinium (MPP^+^) or 6-hydroxydopamine (6-OHDA) reveal that apoptosis is the principal mechanism underlying neuronal death [1-4].

The Akt/glycogen synthase kinase (GSK)-3β/caspase-3 signaling pathways are among the essential components regulating apoptosis [5,6]. Recently, transduction of DA neurons with myristoylated Akt (Myr-Akt), a constitutively active form of Akt prevents 6-OHDA-induced cellular and functional damage of DA neurons [7], Moreover, increased phospho-Akt levels in LINGO-1 knockout mice provide potent protection of DA neurons in 6-OHDA- or N-methyl-4-phenyl-1,2,3,6-tetrahydropyridine neurotoxicity [8]. Several lines of evidence indicate that GSK-3β mediates DA neuronal death in PD animal models produced by MPTP [9] or 6-OHDA [10]. In addition, tau, a well-known substrate for GSK-3β [11,12], mediates DA neuronal death in MPTP-treated mice [9]. Significantly, a number of studies show that caspase-3 contributes to cell death of DA neurons in human PD postmortem brain tissue, animal models of PD, primary cultures of rat mesencephalon, and SN-derived DA cell lines [1,3,4,13].

Gangliosides, sialic acid-containing glycosphingolipids that exist in mammalian cell membranes, are particularly enriched in neuronal membranes [14]. A variety of neurodegenerative disorders are associated with increased ganglioside levels in cerebrospinal fluid (CSF) [15-17]. Furthermore, gangliosides localized to neurofibrillary tangles (senile plaques) in brain tissue of Alzheimer's disease (AD) patients and Betz cells of precentral gyrus in amyotrophic lateral sclerosis patients [18,19]. These findings support the possibility that under pathological conditions, gangliosides serve as cytotoxic factors in the central nervous system (CNS).

Trisialoganglioside, GT1b, one of the major brain ganglioside [14,20], possibly acts as an endogenous regulator in the brain. GT1b induces apoptosis in non-neuronal cells, such as thymocytes [21] and keratinocytes [22]. Moreover, neurotoxic activity of GT1b against DA neurons has been reported in the SN *in vivo *[23] and mesencephalic cultures [24]. However, the mechanisms underlying GT1b-induced DA neuronal death are not clarified.

In this study, we demonstrate for the first time that GT1b neurotoxicity is accompanied by inactivation of Akt, activation of GSK-3β and increase in tau phosphorylation in mesencephalic DA neurons, both *in vivo *and *in vitro*. Our data further confirm that caspase-3 is not a major effector of DA neuronal death in GT1b neurotoxicity.

## Results

### Characterization of GT1b-induced neurotoxicity in neuron-enriched mesencephalic cultures

**Figure 1:**
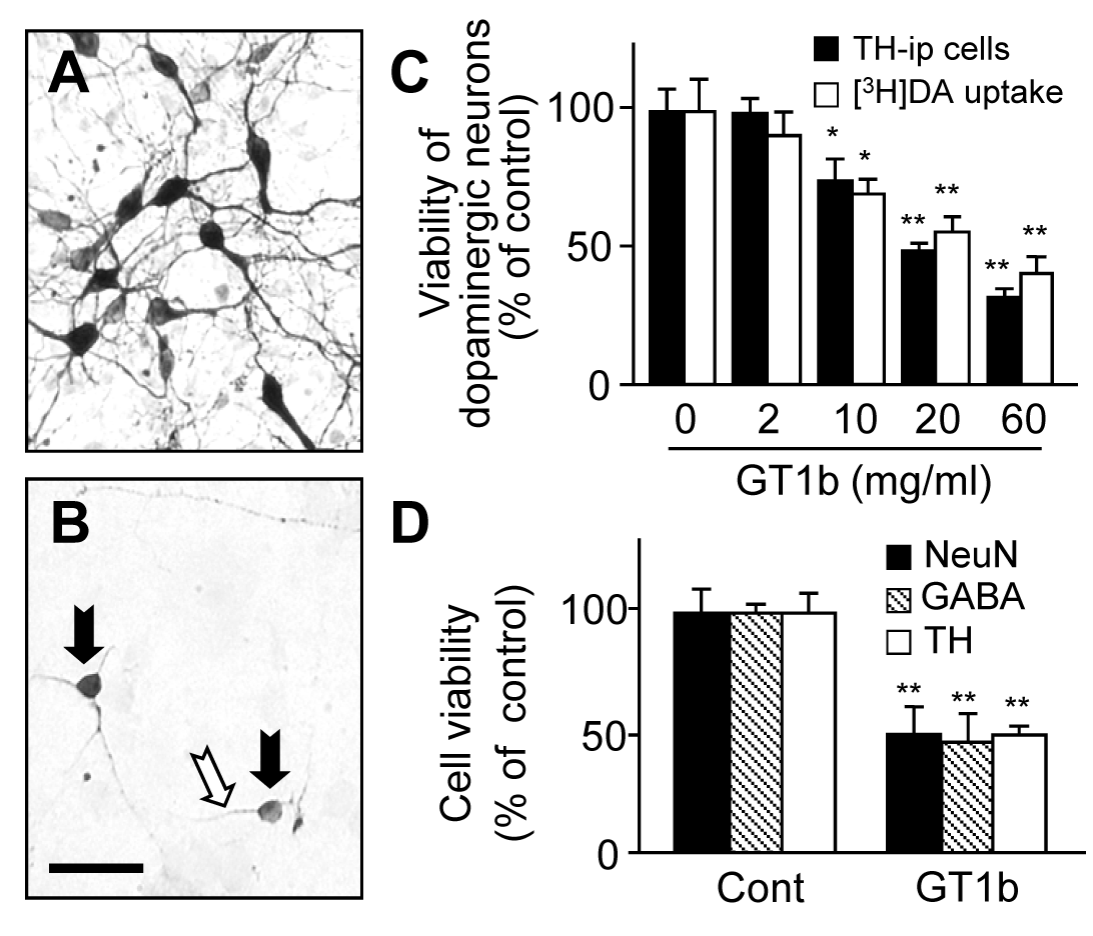
**Neurotoxicity induced by GT1b in neuron-enriched mesencephalic cultures**. (A,B) Cultures were treated with 20 μg/ml GT1b for 24 h and then immunostained with TH antibody. Note a significant loss of TH-ip neurons in mesencephalic cultures following treatment with GT1b (B), compared with untreated control cultures (A). Most of remaining neurons had the short process (white arrows) and rounded and shrunken cell bodies (black arrows). Scale bar, 50 μm. (C) TH-ip neurons were counted or the levels of [^3^H]dopamine uptake were measured in cultures treated with 2-60 μg/ml GT1b for 24 h. The results are expressed as a percentage of the control cultures. (D) Reduction in the number of NeuN-ip, GABA-ip and TH-ip neurons in cultures treated with 20 μg/ml GT1b for 24 h. The values represent the mean ± SEM of triplicate cultures in four separate samples; *P < 0.05, **P < 0.001, significantly different from each control cultures (ANOVA and Student-Newman-Keuls analyses).

Consistent with our previous findings [24], 10-60 mg/ml GT1b significantly attenuated the number of TH-ip neurons by 26-68% and the levels of [^3^H] DA uptake by 31-60% in a dose-dependent manner (Figure 1A,B,C). To selectivity, additional immunostaining was performed with a NeuN antibody for general neurons and GABA antibody for GABAergic neurons (Figure 1D). Treatment with 20 mg/ml GT1b significantly reduced the number of NeuN-ip neurons by 50%, GABA-ip neurons by 54% and TH-ip neurons by 50%, compared with non-treated control cultures.

**Figure 2:**
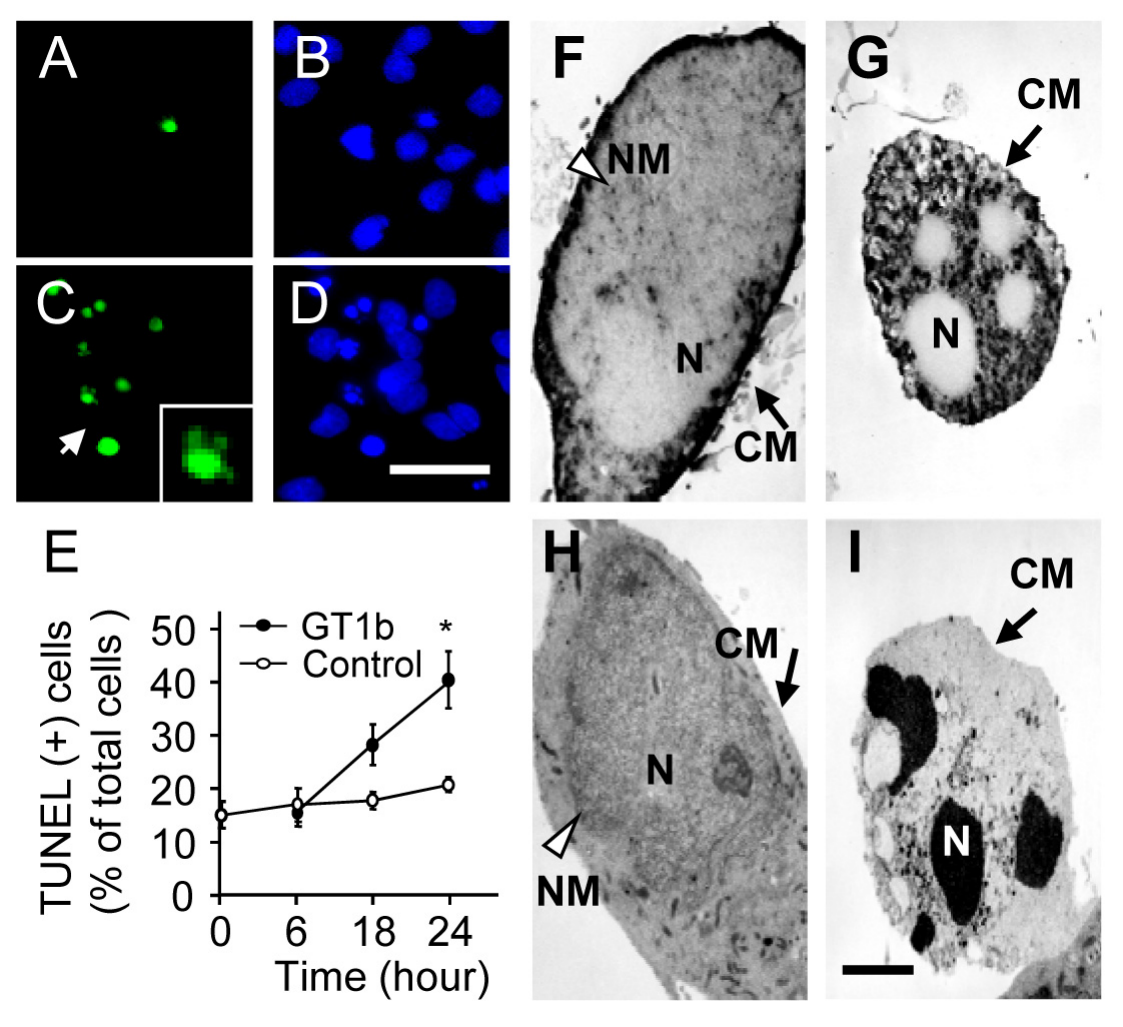
**Neurotoxicity induced by GT1b in neuron-enriched mesencephalic cultures**. (A-D) TUNEL reaction showing DNA fragmentation in the absence (A) or presence (C) of 20 μg/ml GT1b for 24 h in neuron-enriched mesencephalic cultures. B and D, Hoechst 33258 counterstaining of A and C, respectively. Inset, highly magnified cell indicated by arrow. Scale bar, 50 μm. (E) TUNEL (+) cells were counted and expressed as a percentage of Hoechst 33258(+) total cells in control cultures (opened circles) or in cultures treated with 20 μg/ml GT1b (filled circles) at indicated time points. The values represent the mean ± SEM of triplicate cultures in three separate samples; *P < 0.001, significantly different from control cultures (ANOVA and Student-Newman-Keuls analyses). (F-I) Electron microscopic observations showed morphology of neuronal cell death in the absence (F and H) or presence (G and I) of 20 μg/ml GT1b for 24 h. To distinguish DA neurons from non-DA neurons, cultures were immunostained with TH antibody and then electron micrographs taken. Nucleus was well-preserved with fairly intact cell membrane in the control cultures of DA (F) or non-DA (H) neurons. Note that following GT1b, fragmented nuclei were clearly visible in DA (G) or non-DA (I) neurons. CM, cell membrane; N, nucleus; NM, nuclear membrane. Scale bar, 5 μm.

Several morphological and biochemical assays were conducted to further ascertain the mode of cell death and association with caspases. As shown in Figure 2A-D, DNA fragmentation in mesencephalic cultures was confirmed with the TUNEL reaction. The TUNEL-positive (+) cell population was increased in GT1b-treated cultures (Figure 2C), compared with non-treated control cultures (Figure 2A). Hoechst 33258 nuclear staining clearly disclosed condensation and fragmentation of nuclei in GT1b-treated (Figure 2D) but not control cultures (Figure 2B). The percentage of TUNEL (+) cells ranged from 15% to 41%, as determined from the ratio of TUNEL (+) cells to Hoechst 33258 (+) total neuronal cells at the indicated time-points (Figure 2E).

To discriminate between DA and non-DA neurons, mesencephalic cultures were immunostained with a TH antibody specific for DA neurons and electron micrographs obtained. In control cultures, both DA (Figure 2F) and non-DA neurons (Figure 2H) contained intact nuclei with well-preserved cell membranes. In contrast, fragmented nuclei were clearly visible in both DA (Figure 2G) and non-DA neurons (Figure 2I) with a fairly intact cell membrane in GT1b-treated cultures.

### GT1b neurotoxicity is independent of caspase-3

**Figure 3:**
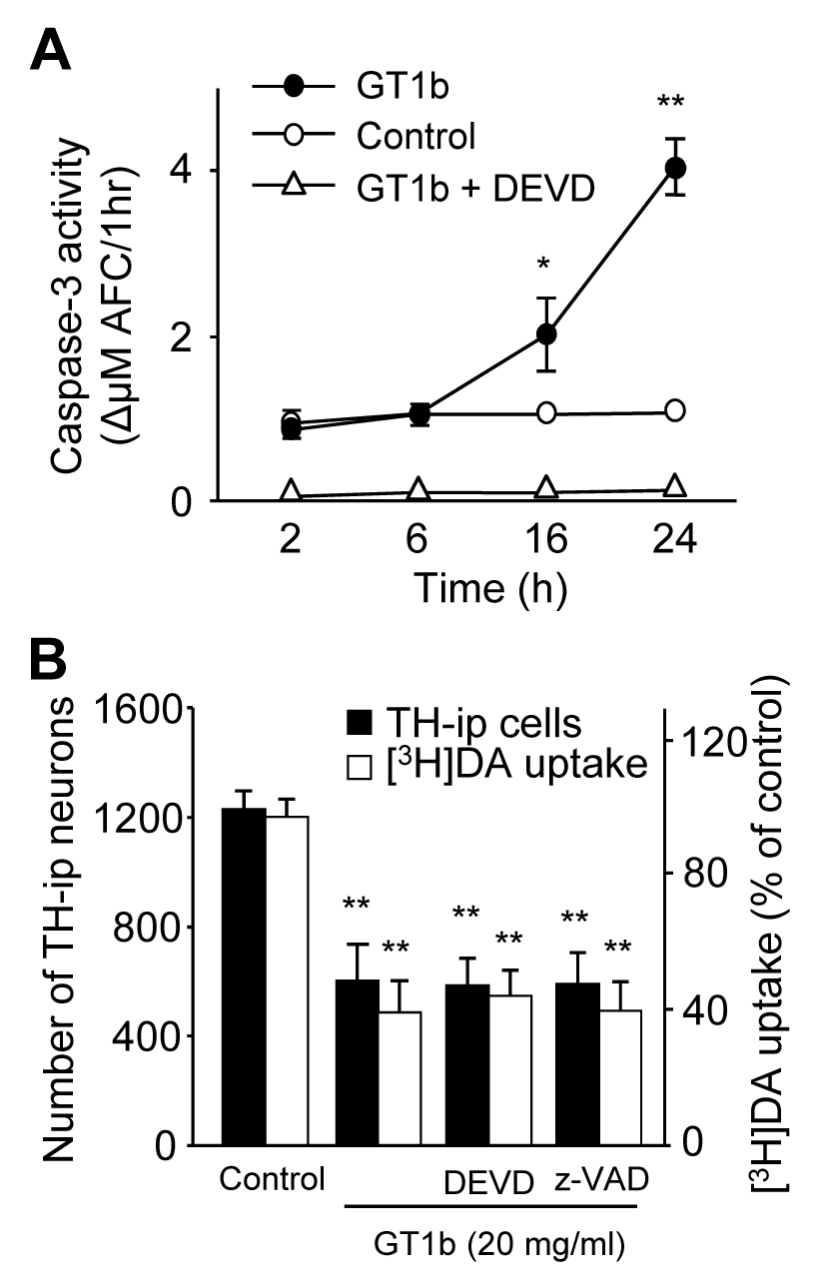
**Neurotoxicity of GT1b was not mediated Caspase-3 in neuron-enriched mesencephalic cultures**. Activity of caspase-3 was measured by DEVD-AFC cleavage (A), and expressed as the amount of Δ AFC per 1 h in cultures treated with 20 μg/ml GT1b in the absence or the presence of caspase-3 inhibitor (DEVD, 10 μM) at the indicated time points. (B) Number of TH-ip neurons and the levels of [^3^H]dopamine uptake were assessed in cultures treated with 20 μg/ml GT1b for 24 h in the absence or the presence of caspase inhibitors, z-VAD (100 μM) or DEVD (10 μM). The values represent the mean ± SEM of triplicate cultures in four separate samples; *P < 0.05, **P < 0.001, significantly different from control cultures (ANOVA and Student-Newman-Keuls analyses).

Accumulating evidence supports the theory that nuclear DNA fragmentation results from activation of caspase-3, which plays a pivotal role in DA neuronal death [3]. Accordingly, to determine whether caspase-3 is involved in GT1b-induced neurotoxicity, cultures were treated with 20 mg/ml GT1b, and caspase-3 activity assessed in cell extracts by monitoring cleavage of its fluorogenic substrate, DEVD-AFC, at indicated time-points. In GT1b-treated cultures, caspase-3 activity was approximately 2 and 4-fold higher than that in control cultures at 16 h and 24 h, respectively (Figure 3A).

Next we examined whether caspase inhibitors block GT1b neurotoxicity. Cultures were treated with a caspase-3-specific inhibitor, DEVD, or a non-specific caspase inhibitor, z-VAD, together with or without GT1b. Surprisingly, neither DEVD nor z-VAD rescued GT1b-induced DA neurotoxicity, as evident from TH-ip neuron counts and level of [^3^H]dopamine uptake (Figure 3B), although the caspase-3-specific inhibitor (DEVD) significantly attenuated caspase-3 activity (Figure 3A). z-VAD or DEVE only as controls had no effects (Additional file 1E). However, DEVD inhibits not only caspase-3 but also caspase-1 and -2. Thus additional experiments were performed using more specific caspase-3 inhibitor, z-DQMD-FMK [25]. Similar to DEVE, z-DQMD-FMK was unable to prevent GT1b neurotoxicity as evidence by TH immunocytochemistry (Additional file 1).

### GT1b promotes inactivation of Akt and subsequent activation of GSK-3β

**Figure 4:**
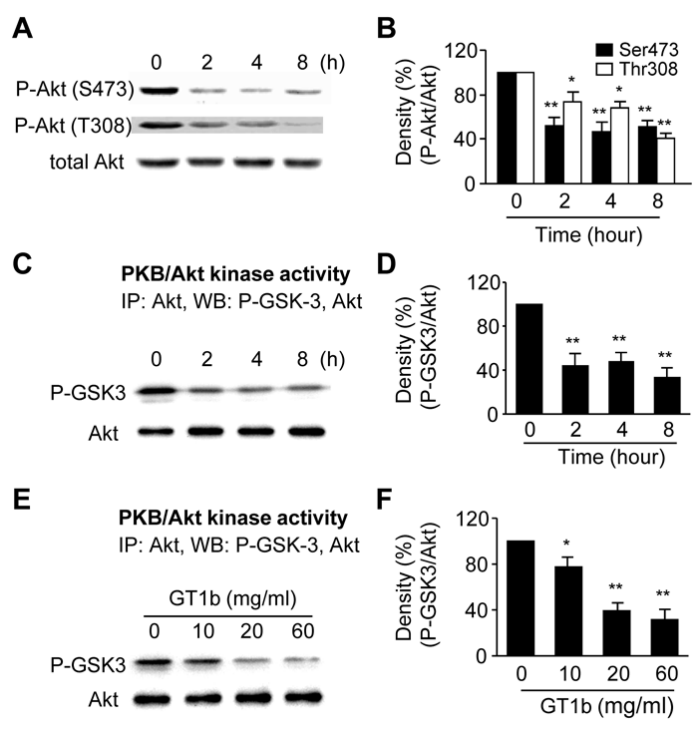
**Reduction in phosphorylation/activation of Akt by GT1b**. (A) Phosphorylation of Akt was detected by immunoblotting with phospho-Akt (Ser473) and phospho-Akt (Thr308) antibody in cultures treated with 20 μg/ml GT1b at indicated time points. (B) The histogram shows quantification of phospho-Akt levels. (C-F) The activity of Akt was measured using immunoprecipitated Akt, and then it was mixed with the GSK-3α/β fusion protein (1 μg/assay). Phosphorylated GSK-3α/β was detected by immunoblotting with phospho-GSK-3α/β antibody. (D,F) The histograms show quantification of phospho-GSK-3α/β levels of C and E, respectively. The values represent the mean ± SEM of four to five separate experiments; *P < 0.01, **P < 0.001, significant from non-treated control cultures (0 h) (ANOVA and Student-Newman-Keuls analyses).

The Akt signaling pathway is essential for the survival of various cell types [26,27], including DA neurons [7]. Specific PD-related pathological challenges by dephosphorylation/inactivation of Akt and dephosphorylation/activation of its downstream substrate, GSK-3β may be associated with MPP^+^- or 6-OHDA-induced neurotoxicity [10,28]. To examine the involvement of Akt in GT1b neurotoxicity, cultures were treated with 20 mg/ml GT1b, and Western blotting analysis performed using phospho-Akt (Ser473) or phospho-Akt (Thr308) antibody at the indicated time-points. Decrease in Akt phosphorylation at both Ser473 and Thr308 was detected as early as 2 h, and sustained up to 8 h in GT1b-treated cultures (Figure 4A,B). Additionally, Akt kinase activity was measured by assessing phosphorylation of its substrate, GSK-3α/β fusion protein by immunoprecipitated Akt. Akt kinase activity, quantified and expressed as a percentage of phosphorylated GSK-3α/β to total Akt, was inhibited by 56% as early as 2 h after the application of 20 μg/ml GT1b, compared to control cultures (0 h) (Figure 4C,D). GT1b (10-60 μg/ml) significantly attenuated Akt kinase activity (by 22-68%) at 6 h post-treatment, compared with non-treated control cultures (Figure 4E,F).

**Figure 5:**
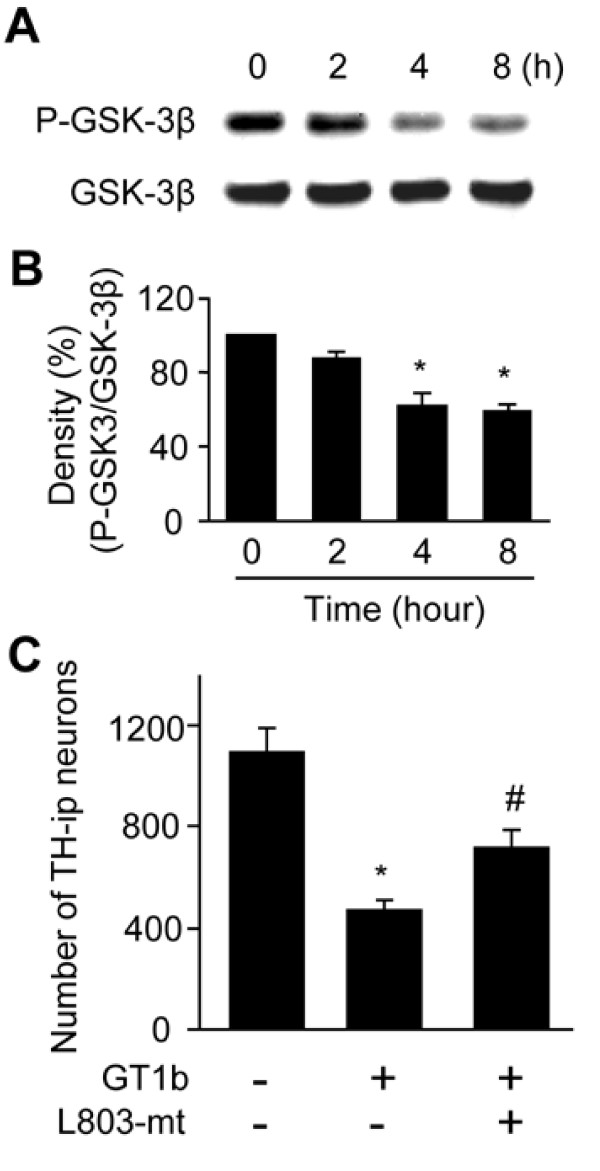
**GT1b increases the activity GSK-3β by dephosphorylation in neuron-enriched mesencephalic cultures**. (A) Western blot analysis showing dephosphorylation of GSK-3β in GT1b-treated cultures. Cell lysates were analyzed by Western blot analysis with phospho-GSK-3β (Ser9) antibody at indicated time points. After membrane stripping, blots were reprobed with total GSK-3β. (B) The histogram shows quantification of phospho-GSK-3β levels. The values represent the mean ± SEM of four separate experiments; *P < 0.001, significant from non-treated control cultures (0 h) (ANOVA and Student-Newman-Keuls analyses). (C) TH-ip neurons were counted in cultures treated with 20 μg/ml GT1b for 24 h in the absence or presence of 20 μM L803-mt, GSK-3β specific inhibitor. The values represent the mean ± SEM of triplicate cultures in three to five separate samples. *P < 0.001, significant from non-treated control cultures; ^#^*P *< 0.01, significant from GT1b-treated cultures (ANOVA and Student-Newman-Keuls analyses).

GSK-3β is a major substrate of Akt. Physiologically, activated Akt inhibits GSK-3β activity by enhancing phosphorylation at Ser9, consequently promoting cell survival [29]. Accordingly, we hypothesize that the decrease in phospho-Akt, in turn, reduces GSK-3β phosphorylation, leading to its activation and eventual cell death. To examine this theory, Western blotting analysis was performed using a phospho-GSK-3β (Ser9) antibody. As shown in Figure 5A, GSK-3β phosphorylation was decreased by 12-41% at specific time-points, compared with control cultures (0 h) (Figure 5B).

To assess whether suppression of GSK-3β activity blocks GT1b neurotoxicity, cultures were treated with the GSK-3β-specific inhibitor, L803-mt (20 μM) [10], together with GT1b for 24 h. L803-mt rescued TH-ip neuron death by 53% in relation to GT1b-treated cultures (Figure 5C). However, L803-mt alone had no effect (data not shown).

### GT1b induces tau hyperphosphorylation through GSK-3β activation

**Figure 6:**
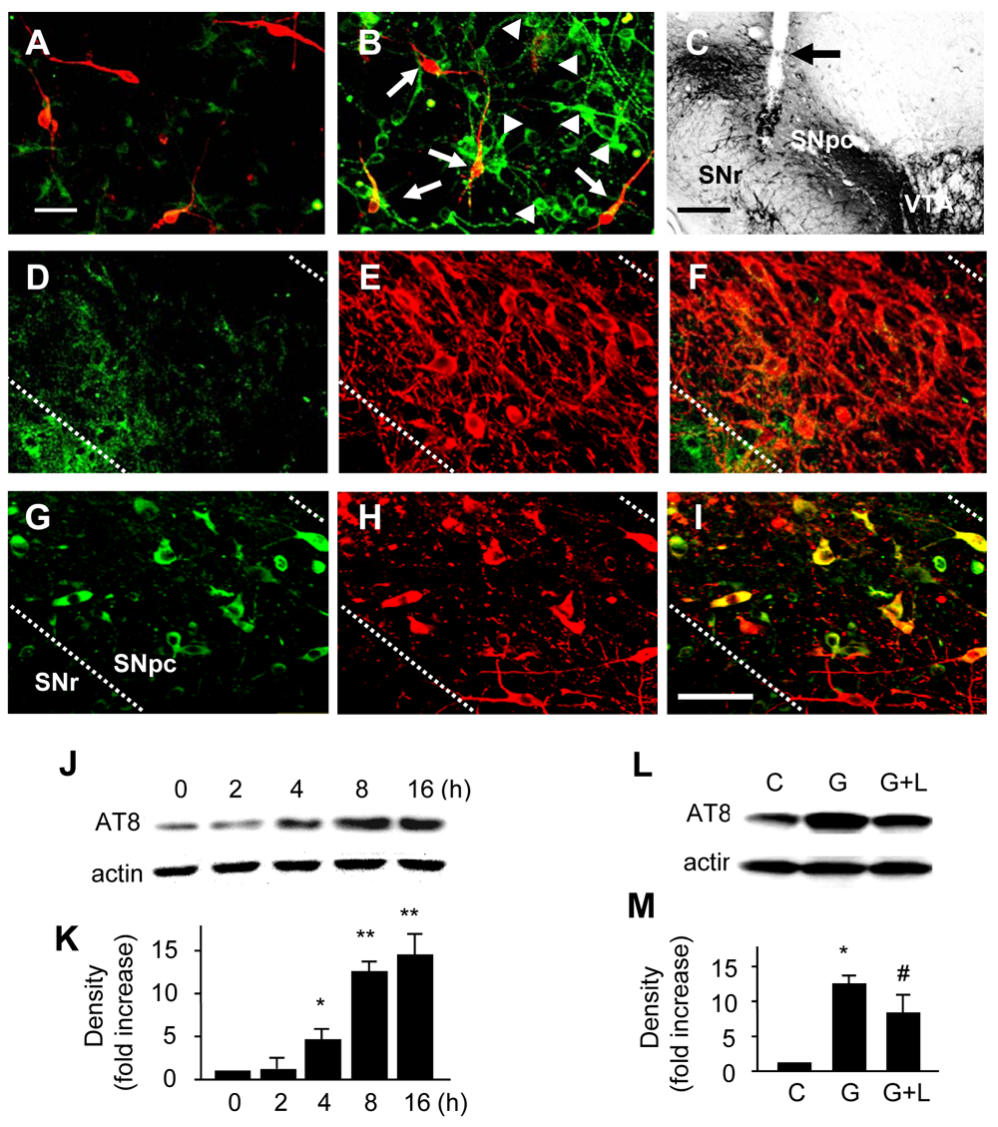
**Phosphorylation of tau is increased by GT1b treatment in mesencephalic cultures**. Double-immunostaining with AT8 (Ser202; green)) for phospho-tau and TH (red) for DA neurons in mesencephalic cultures treated with vehicle (A) or 20 μg/ml GT1b (B) for 8 h. (D-I) Double-immunostaining showed Tau hyperphosphorylation in the GT1b-injected SN. SN tissues were prepared 72 h after PBS (D-F) or GT1b (60 μg/3 μl, G-I) injection, processed for immunostaining with TH antibody alone (C), double-immunostaining with AT8 (D, G, green) and TH (E, H, red) antibody, and then both images were merged (F, I). Scale bar, 10 μm in A-C, 200 μm in F and 50 μm G-I. SNpc, substantia nigra pars compacta; SNr, substantia nigra reticulata; VTA, ventral tegmental area. (J,K) Cell lysates were analyzed by Western blot analysis with AT8 (Ser202) antibody at indicated time points. After membrane stripping, blots were then reprobed with actin antibody. The histogram shows quantitation of tau phosphorylation. (L.M) Tau phosphorylation is reversed by L803-mt, GSK-3 inhibitor in GT1b-treated mesencephalic cultures. The histogram shows quantitation of tau phosphorylation. The values represent the mean ± SEM of triplicate cultures in three to five separate samples. *P < 0.001, **P < 0.01 significant from non-treated cultures; ^#^*P *< 0.01, significant from GT1b-treated cultures (ANOVA and Student-Newman-Keuls analyses). C, control; G, GT1b; L, L803mt.

Tau is phosphorylated by GSK-3β activated as a result of Akt inactivation [6]. Earlier studies show that hyperphosphorylation of tau inhibits its binding to microtubules, resulting in neuronal cell death [30]. Accordingly, we examined whether GT1b enhances tau phosphorylation. Cultures were treated with vehicle as controls (Figure 6A) or 20 ug/ml GT1b for 8 h (Figure 6B) and double-immnuostained with the AT8 antibody for phospho-tau (Ser202, Green) and TH antibody for DA neurons (Red). The results revealed that following GT1b treatment, AT8-ip-phosphorylated tau was localized within both TH-ip (arrow) and non-TH-ip neurons (arrowhead) with strongly stained soma and processes (Figure 6B), compared to control (Figure 6A). Consistent with our previous results [23], intranigral injection of GT1b induced degeneration of TH-ip neurons in the ipsilateral SN (Figure 6C), compared with the contralateral side (data not shown) at 72 h post-treatment. Additional double immunostaining using SN tissues revealed that phosphorylated tau (Figure 6G, green) and TH-ip neurons (Fg.6G, red) co-localize in GT1b-injected SN (Figure 6I, yellow). Few AT8-ip neurons were visible in PBS injected SN as controls (Figure 6D-F).

Additional Western blot analyses disclosed that 20 μg/ml GT1b significantly upregulated tau phosphorylation in a time-dependent manner (Figure 6J,K). To assess whether suppression of GSK-3β activity affects the tau hyperphosphorylation level, cultures were treated with a GSK-3β specific inhibitor, L803-mt, together with GT1b for 16 h. L803-mt reversed tau hyperphosphorylation by 47%, compared to GT1b-treated cultures (Figure 6L,M).

## Discussion

Increasing evidence has shown that gangliosides enhance cell death in various cell types. For example, GM1 and GT1b produce an increase of cell death in feline thymocytes [21]. GM3 causes apoptosis, decreases BrdUrd incorporation, and up-regulates the cdk inhibitor p27 ^kip ^in proliferating astrocytes [31]. Many studies also demonstrate that, GD3 disrupts mitochondria membrane potential [32,33], induces cell death and activates caspases in HuT78, derived from a human cutaneous T cell lymphoma [34]. Similarly, neurotoxicity of major brain gangliosides, such as GD1a and GD1b against dopaminergic neurons in mesencephalic cultures has been reported [35]. We also found that GT1b was neurotoxic to mesencephalic dopaminergic neurons *in vivo *and *in vitro *[23,24]. This is consistent with our present findings that GT1b neurotoxicity produces DNA cleavage as well as condensation and fragmentation of nuclei, as evident from the TUNEL reaction, Hoechst 33258 nuclear staining and immunoelectron microscopic observations, respectively.

### GT1b neurotoxicity is not mediated by caspase-3

Nuclear DNA damage occurs due to activation of caspase-3, which appears to be a major effector of DA neuronal cell death in human PD patients [1], animal models of PD [4], and MPP^+^-treated rat mesencephalic cultures [13] and SN-derived DA cell lines [36]. Regarding this, our data indicated that GT1b-induced nuclear DNA damage was accompanied by caspase-3 activation, which was effectively suppressed by the specific inhibitor, DEVD (Figure 3A). However, the caspase-3 inhibitor, DEVD, and the non-specific caspase inhibitor, Z-VAD, were unable to block GT1b-induced neurotoxicity, as confirmed from the number of TH-ip neurons and level of [^3^H]dopamine uptake in mesencephalic cultures (Figure 3B). These results carefully suggest that under our experimental conditions, caspase-3 may not be a major effector of GT1b-induced DA neuronal cell death. This hypothesis is supported by recent findings that inhibition of caspases, including caspase-3, does not always prevent neuronal death in MPP^+^-treated cerebellar granule neurons [37], proteasome inhibitor-mediated death of HT4 cells (hippocampal cell line) [38], and global ischemia-induced hippocampal neurodegeneration [39]. Moreover, a wide range of caspase inhibitors, including DEVD, had no effects on cytotoxicity induced by another major brain ganglioside, GD3, in rat hepatocytes [32] and HuT78, a cell line derived from a human cutaneous T cell lymphomas [34].

### The Akt/GSK-3β/tau signaling pathway is implicated in GT1b neurotoxicity

Akt, a serine/threonine protein kinase, is significantly associated with neuronal survival/degeneration [5,26]. In particular, decreased Akt signaling is associated with neurodegeneration in the brains of MPTP [40,41] or 6-OHDA-induced Parkinsonism [42] and *Drosophila *models of PD [43]. Moreover, several studies demonstrate that transduction of DA neurons with myristoylated Akt (Myr-Akt), [7] and increasing phospho-Akt in LINGO-1 knockout mice [8] provide potent protection against 6-OHDA- or MPP^+^-induced cellular and functional damage of DA neurons, respectively, consistent with our present observation that GT1b neurotoxicity is associated with dephosphorylation/inactivation of Akt. The result is further supported by previous findings that GT1b-induced apoptosis is mediated by decreased phosphorylation of Akt in keratinocyte-derived SCC12 cells [44], and GT1b depletion increases cell survival through Akt activation [22].

Akt contains two regulatory phosphorylation sites, Thr308 and Ser473. While phosphorylation at both sites is dependent on phosphoinositide 3-OH kinase (PI3K), the mechanism of Ser473 phosphorylation remains controversial. In this regard, there is evidence suggesting that the Ser473 site is autophosphorylated [45,46] or phosphorylated by distinct serine kinase, including the integrin-linked kinase (ILK) [27,47]. In SSC12 cells, GT1b-induced cell death is mediated by inhibiting phosphorylation of Akt at Ser473, but not Thr308, through inactivation of ILK that does not require PI3K [22,44]. Data from the present study show that GT1b neurotoxicity is accompanied by Akt dephosphorylation at both Ser473 and Thr308. Therefore, it is likely that in mesencephalic cultures, ILK or PI3K signaling or both participate in GT1b neurotoxicity. However, we did not provide direct evidence of whether GT1b neurotoxicity is mediated by ILK or PI3K signaling or both.

The anti-apoptotic function of Akt involves phosphorylation/inactivation of its downstream substrate, GSK-3β. Inactivation of Akt triggers GSK-3β activity through decreasing phosphorylation, which plays a key role in neuronal loss occurring in neurodegenerative diseases, such as PD [9,48] and AD [6]. Two PD mimetics, 6-OHDA and MPP^+^, induce GSK-3β-dependent neurodegeneration in cell types, including SH-ST5Y, PC12, and cerebellar granule neurons [10,28], suggesting that GSK-3β is a key mediator of neuronal death. Our results show that in mesencephalic cultures, GT1b induces dephosphorylation of GSK-3β, indicative of GSK-3β β involvement. This finding is further supported by TH immunocytochemistry data showing that blockage of GSK-3β activation by the inhibitor, L803-mt, leads to reduced GT1b neurotoxicity.

**Figure 7:**
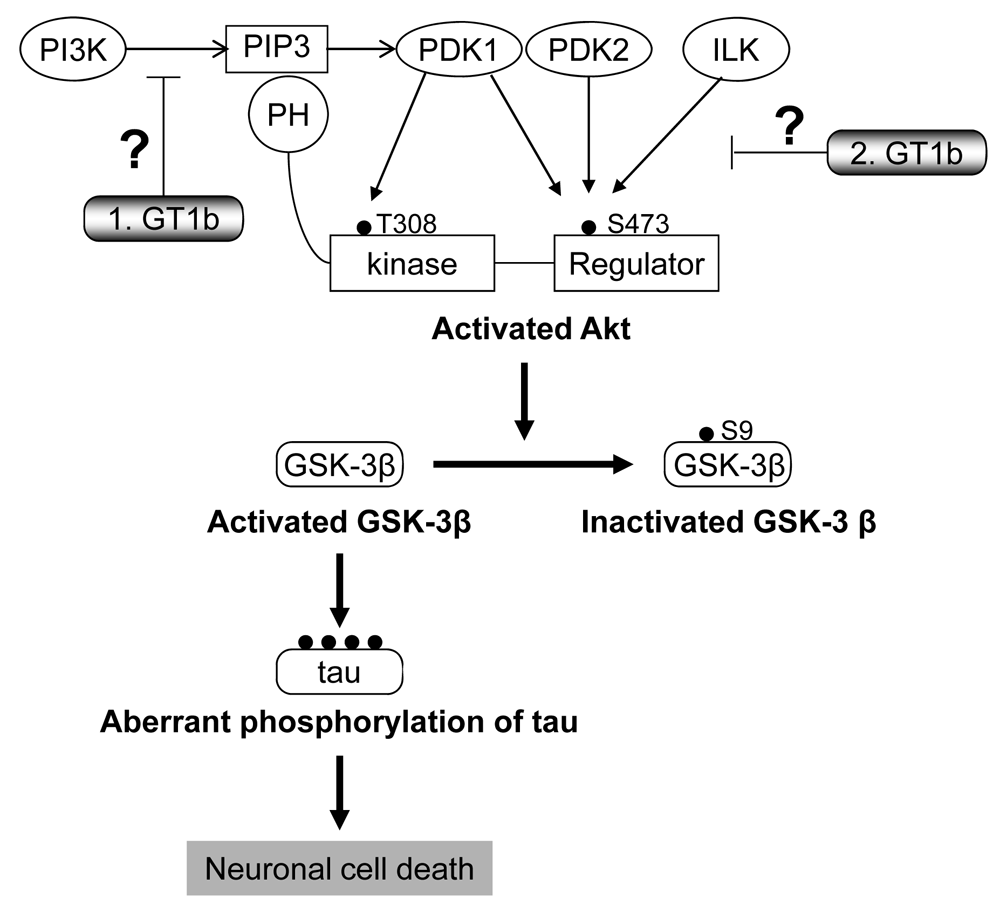
**Schematic drawing of Akt/GSK-3β/tau signaling**. Akt is activated in response to ILK or PI3 Kinase signaling, and subsequent activation of Akt. Akt is activated in response to ILK or PI3 kinase signaling, and subsequent activation of Akt. Activated Akt phosphorylates GSK-3β at Ser 9, leading to GSK-3β inactivation. Akt inactivation by GT1b induces dephosphorylation of GSK-3β, leading to GSK-3β activation. GSK-3β activation results in aberrant tau phosphorylation, axon instability, and neuronal death. Although we did not provide direct evidence of whether GT1b neuroxocicity is mediated by ILK or PI3K signaling or both, participation of GT1b in inhibition of Akt upstream signals, such as ILK or PI3K, is predicted.

Our results are comparable with recent reports showing that suppression of GSK-3β β activity with selective inhibitors, such as indirubin-3'-oxime and AR-A014418, prevents MPTP-induced loss of DA neurons *in vivo *[9], and novel synthetic inhibitors for GSK-3β protect DA neurons against MPP^+ ^toxicity in mesencephalic cultures via suppression of α-synuclein protein expression [48]. These results collectively imply that GT1b induces DA neuronal death through the Akt/GSK-3β signaling pathway (Figure 7).

Tau is one of the major microtubule-associated proteins identified in neurons. Hyperphosphorylation of tau inhibits its binding to microtubules, leading to neuronal death [49-51] and is mediated by several protein kinases, including GSK-3β [12]. Our Western blot and double immunostaining data show that in parallel with increased GSK-3β activity, GT1b induces tau hyperphosphorylation in mesencephalic DA neurons *in vivo *and *in vitro*. In addition, L803-mt, a GSK-3β inhibitor, attenuates tau phosphorylation, suggesting that GT1b neurotoxicity is mediated by the GSK-3β/tau signaling pathway.

## Conclusions

In the present study, we show that GT1b neurotoxicity is associated with inhibition of Akt, resultant activation of GSK-3β and increasing cleavaged tau as well as phosphorylation in mesencephalic neurons including DA neuron. Interestingly, while GT1b activates caspase-3, a key molecule of DA neuronal death, inhibition of caspase-3 fails to rescue DA neurons. Our data collectively suggest that GT1b neurotoxicity is associated with Akt/GSK-3β/tau, but not with caspase-3.

## Methods

### Materials

Materials were purchased from the following companies: mouse anti-tyrosine hydroxylase (TH; Pel-Freez, Rogers, AR, USA), rabbit anti-tyrosine hydroxylase (TH; Pel-Freez), mouse anti-neuron specific nuclear protein (NeuN; Chemicon, Temecula, CA, USA), rabbit anti-GABA and mouse anti-actin (Sigma, St. Louis, MO, USA), rabbit anti-phospho-Akt (Ser473), rabbit anti-phospho-Akt (Thr308), rabbit anti-phospho-GSK-3β (Ser9), rabbit anti-Akt and rabbit anti-GSK-3β (Cell Signaling Technology, MA, USA), mouse anti-AT8 (phospho-tau (Ser202), INOGENETICS, Gent, Belgium), biotinylated secondary antibody and ABC kit (Vector Laboratory, Burlington, CA, USA), FITC-conjugated anti-mouse IgG and Texas Red-conjugated anti-rabbit IgG (Molecular Probes, OR, USA). GT1b (Matreya, Pleasant Gap, PA), DEVD-FMK (Biochemicals, San Diago, CA), z-DQMD-FMK (Tocris Elliscille, MO), z-VAD (R&D systems Minneapolis, MN), L803-mts (Calbiochem, San Diego, CA, USA), tritiated dopamine ([^3^H]dopamine; Amersham, Oakville, ON, USA), Apotag fluorescein *in situ *detection kit (Chemicon), Hoechst 33258 (Molecular Probes), ApoAlert Caspase Fluorescent Assay Kits (CLONTECH Laboratories, Palo Alto, CA, USA), Akt kinase Assay Kit (Cell Signaling Technology).

### Mesencephalic cultures

Neuron-enriched mesencephalic cultures were prepared as previously described with some modifications [24]. In brief, cells from the ventral mesencephalons of embryonic day 14 Sprague-Dawley rats were seeded on 12 mm round aclar plastic coverslips or culture plates pre-coated with 100 μg/ml poly-D-lysine (Sigma) and 4 μg/ml laminin (Upstate Biotech, NY, USA) at a density 1.25~2 × 10^5 ^cells/cm^2^. The cultures were incubated in a humidified incubator at 37°C, 5% CO_2 _for 24 h. To suppress the proliferation of glial cells, on the second day *in vitro *(DIV2), the media were replaced with a chemically defined serum-free media (DM) composed of Ham's nutrient mixture F12/DMEM and supplemented with ITS (insulin, transferrin and sodium selenite; Sigma), glucose, L-glutamine and penicillin/streptomycin (P/S). On DIV 4, cultures were treated with various concentrations of GT1b in DM without ITS. As described previously, the cell composition included ~5% astrocytes and less than 1% microglia which were glial fibrillary acidic protein (GFAP; Sigma) immunopositive (ip) and CD11b (OX-42, Serotec, Oxford, UK)-ip cells, respectively. The remaining cells were presumed to be neurons, 4.5~6% and 9.5~11% of which were tyrosine hydroxylase-ip (TH-ip) and gamma amino butyric acid (GABA, ; Sigma)-ip neurons, respectively [35].

### Immunocytochemistry

As previously described [24,35], paraformaldehyde-fixed cells were immunostained with following cell type specific antibodies; mouse-neuron specific nuclear protein (NeuN, 1:300) for general neurons, mouse-TH (1:7500) for DA neurons or rabbit-GABA (1:2000) for GABAergic neurons. Cultures were incubated with a primary antibody for overnight at room temperature (RT) and subsequently incubated with an appropriated biotinylated secondary antibody. Immunostaining was visualized by the ABC method followed by color development with diaminobenzidine (DAB; Sigma), and analysed under a bright-field microscope (Olympus, Tokyo, Japan).

### Measurement of Dopamine Uptake

The measurement of dopamine uptake was performed as our described previously [24,35]. Briefly, cultures were washed twice with the incubation solution (HBSS containing 10 mM HEPES, 0.6% glucose, 0.2 mM pargyline, and 0.01% ascorbic acid, pH 7.4), and then incubated at 37°C for 20 min in incubation solution with a final concentration of 83.3 nM tritiated dopamine ([^3^H]dopamine, 444 GBq/mmol). Blank values were obtained by incubating cultures at 0°C. The reaction was terminated by removal of the solution followed by three rapid washes with ice-cold incubation solution. The cultures were then lysed with lysis buffer (0.2 M NaOH containing 0.2% Triton X-100) and transferred to scintillation vials for counting.

### TUNEL assay

The TUNEL assay was performed using the Apotag fluorescein *in situ *detection kit that detects the 3'-OH region of cleaved DNA. Briefly, cultures were exposed to GT1b 20 μg/ml for indicated time points. And then cultures were fixed with 4% paraformaldehyde in PB for 15 min at RT. Subsequently, cultures were incubated with a mixture of terminal deoxynucleotidyl transferase and reaction buffer containing FITC fluorescein conjugated-digoxigenin-dUTP in a humidified chamber for 1 h at 37°C, and washed in washing buffer for 10 min. Finally, cultures were counterstained with 1 μg/ml Hoechst 33258 at RT for 10 min, and viewed using an Olympus IX71 confocal laser scanning microscope (Olympus, Tokyo, Japan).

### Immuno-electron microscopy

Mesencephalic cultures were fixed using Karnovsky's fixative solution (2% paraformaldehyde, 2% glutaraldehyde, 0.5% calcium chloride in cacodylate buffer, pH 7.2) for 30 min and then immunostained with mouse-TH antibody. Subsequently, cultures were incubated with a biotinylated anti-mouse IgG. Immunostaining was visualized by the ABC method followed by color development with DAB. Immunostained cultures were processed for an electron microscope. In brief, cultures were washed with cacodylate buffer, dehydrated in a series of graded ethanol, and then embedded in epon mixture. Ultrathin sections were cut using on Reichert Jung Ultracut S (Leica, Markham, Ontario, Canada) and mounted on grids, stained with uranyl acetate and lead citrate, and analyzed under a Zeiss EM 902 A electron microscope (Carl Zeiss, German).

### Measurement of caspase activities

Caspase activity was determined using the ApoAlert Caspase Fluorescent Assay Kits in mesencephalic cultures. Briefly, cultures were treated with 20 μg/ml GT1b for indicated time points. And then the harvested lysate was centrifuged for 20 min at 14,000g and the supernatant (10 μg/ml) were incubated at 37°C in a reaction buffer adding 10 mM DTT with the fluorogenic substrate DEVD-AFC, and the emitted fluorescence was measured in a Spectrometer-Luminescene (Perkin Elmer, Norwalk, CT, USA).

### Akt kinase activation

For Akt kinase assay, cultures were washed with ice-cold phosphate-buffered saline (PBS), lysed with cell lysis buffer (20 mM Tris-HCL, pH 7.5, 150 mM NaCl, 1 mM EDTA, 1 mM EGTA, 1% Triton X-100, 2.5 mM sodium pyrophosphate, 1 mM β-glycerophosphate, 1 mM Na_3_VO_4_, 1 μg/ml leupeptin; Cell Signaling Technology) adding 1 mM phenylmethylsulfonyl fluoride (PMSF). Equivalent amounts of Akt were immunoprecipitated by rabbit-Akt antibody prebound to protein A-agarose beads, and kinase assays were carried out according to the manufacturer's instruction manual of the Akt kinase Assay Kit (Cell Signaling Technology). GSK-3α/β fusion protein was used as the substrate for Akt. Immunoprecipitated Akt and phosphorylated GSK-3α/β were measured by Western blotting using Akt antibody and phospho-GSK-3α/β (Ser 21/9) antibody, respectively.

### Western blot analysis

Cultures were washed with ice-cold PBS, lysed with cell lysis buffer adding 1 mM PMSF and protease inhibitor mixture (Sigma). Equal amounts of protein (50 μg) were mixed with loading buffer (0.125 M Tris-HCl, pH 6.8, 20% glycerol, 4% SDS, 10% mercaptoethanol, and 0.002% bromophenol blue), boiled for 5 min, and separated by SDS-PAGE. After electrophoresis, proteins were transferred to polyvinylidene difluoride (PVDF) membranes (Millipore, Bedford, MA, USA) using an electrophoretic transfer system (Bio-Rad, Hercules, CA, USA). The membranes were then incubated for overnight at 4°C with one of the following the specific primary antibodies: rabbit-phospho-Akt (Ser473), rabbit-phospho-Akt (Thr308), rabbit-phospho-GSK-3β (Ser9), and mouse-AT8 phospho-tau (Ser202). After washing, the membranes were incubated with HRP-conjugated secondary antibodies (1:2000; Amersham Biosciences) for 1 h at RT. Finally, the blots were developed with enhanced chemiluminescence detection reagents. The blots were reprobed with antibodies against rabbit-Akt, rabbit-GSK-3β r mouse-actin (1:10000). For semiquantitative analyses, the densities of bands on immunoblots were measured with the Computer Imaging Device and accompanying software (Fujifilm, Tokyo, Japan).

### Infusion of GT1b and tissue preparation

For infusion of GT1b in the SN, female SD rats (230~250g) were anesthetized with an injection of chloral hydrate [360 mg/kg, intraperitoneal (i.p.) injection], positioned in a stereotaxic apparatus (Kopf Instrument, Tujunga, CA, USA). Each rat received a unilateral administration of GT1b into the right SN [anteroposterior (AP) 5.3 mm, mediolateral (ML) 2.3 mm, dorsoventral (DV) 7.6 mm from bregma], according to the atlas of Paxinos and Watson (1998). All injections were made using a Hamilton syringe equipped with a 30S-gauge beveled needle and attached to a syringe pump (KDScientific, MA, USA). Infusions were made at a rate of 0.2 μl/min for GT1b (60 μg in 3 μl distilled water). Animals were transcardially perfused with saline solution containing 0.5% sodium nitrate and heparin (10 U/ml) and fixed with 4% paraformaldehyde dissolved in 0.1 M PB. Brains were removed from the cranium, post-fixed for 1 h, washed in 0.1 M PB and immersed in 30% sucrose solution until they sank. Tissues were sectioned at a thickness of 35 μm using a sliding microtome. Every sixth serial section was selected and processed for immunohistochemical staining.

### Double-immunostaining

For immunofluorescence double labeling, cultures or tissue sections were incubated in a combination of mouse-AT8 antibody and rabbit-TH antibody. The next day, cultures or tissue sections were rinsed and incubated with FITC-conjugated anti-mouse IgG (1:200) and Texas Red-conjugated anti-rabbit IgG (1:200) for 1 h at RT. Stained cells were viewed using an Olympus IX71 confocal laser-scanning microscope (Olympus).

### Statistical analysis

All data are represented as the means ± SEM. The statistical significance of differences was assessed using analysis of variance (ANOVA), followed by Student-Newman-Keuls analyses or Student's two-tailed t-test (SPSS for windows: Standard Version). Statistical significance was defined as *p *< 0.05 for all analyses.

## Abbreviations

6-OHDA: 6-hydroxydopamine; AD: Alzheimer's disease; ALS: amyotrophic lateral sclerosis; DA: dopaminergic; DIV: day in vitro; GFAP: glial fibrillary acidic protein; GSK-3β: glycogen synthase kinase 3; GT1b: trisialoganglioside 1b; ip: immunopositive; PD: Parkinson's disease; SN: substantia nigra; TH: tyrosine hydroxylase; TUNEL: transferase-mediated fluorescein-dUTP nick-end labeling.

## Authors' contributions

EC carried out mesencephalic culture, animal surgery and sample preparation, participated in the western blot analysis, immunohistochemistry, statistical analysis and drafted the manuscript. EB, carried out mesencephalic culture, animal surgery and sample preparation, participated in immunohistochemistry, and statistical analysis and revised the manuscript. SS performed immune-electron microscopy. SS, YL, HB contributed to analysis, programming tools and helped in the interpretation of the data. BJ assisted in study conceptualization, design and manuscript write-up. All authors read and approved the final manuscript.

## Supplementary Material

Additional file 1 **Neurotoxicity of GT1b was not mediated Caspase-3 in neuron-enriched mesencephalic cultures**. (A-D) Cultures were treated with 20 μg/ml GT1b for 24 h in the absence or the presence of specific caspase-3 inhibitors, DQMD-FMK, and then immunostained with TH antibody. Note a significant loss of TH-ip neurons in mesencephalic cultures following treatment with GT1b in the absence (B) or the presence of DQMD-FMK 20 uM (C), compared with untreated control cultures (A). (D) The number of TH-ip neurons was counted. Note that z-DQMD-FMK didn't reduce neurotoxicity of GT1b in 1 ~ 20 uM range. 40 uM of z-DQMD-FMK had toxic effect in mesencephalic neurons about 40% compare with controls. Scale bar, 50 μm. (E) The levels of [^3^H]dopamine uptake were assessed in cultures treated with z-VAD (100 μM) or DEVD (10 μM) for 24 h. The values represent the mean ± SEM of duplicate cultures in three separate samples; *P < 0.01, **P < 0.001, significantly different from control cultures (ANOVA and Student-Newman-Keuls analyses). Click here for file
